# Investigation of potential protein biomarkers for the screening of placental-mediated fetal growth restriction disorders using targeted proteomics Olink technology

**DOI:** 10.3389/fimmu.2025.1542034

**Published:** 2025-05-16

**Authors:** Xinyao Zhou, Wuqian Wang, Luan Chen, Yingjun Yang, Xing Wei, Jia Zhou, Kuan Sun, Ping Tang, Xiaofang Sun, Shengying Qin, Luming Sun

**Affiliations:** ^1^ Department of Fetal Medicine & Prenatal Diagnosis Center, Shanghai Key Laboratory of Maternal Fetal Medicine, Shanghai Institute of Maternal-Fetal Medicine and Gynecologic Oncology, Shanghai First Maternity and Infant Hospital, School of Medicine, Tongji University, Shanghai, China; ^2^ Department of Obstetrics and Gynecology, Guangdong Provincial Key Laboratory of Major Obstetric Diseases, Guangdong Provincial Clinical Research Center for Obstetrics and Gynecology, Guangdong-Hong Kong-Macao Greater Bay Area Higher Education Joint Laboratory of Maternal-Fetal Medicine, The Third Affiliated Hospital, Guangzhou Medical University, Guangzhou, China; ^3^ Bio-X Institutes, Key Laboratory for the Genetics of Developmental and Neuropsychiatric Disorders (Ministry of Education), Shanghai Jiao Tong University, Shanghai, China; ^4^ Department of Obstetrics, Center of Fetal Medicine & Intrauterine Pediatrics, Xinhua Hospital, Shanghai Jiao Tong University School of Medicine, Shanghai, China; ^5^ Jiaxing Maternity and Children Health Care Hospital, Affiliated Women and Children Hospital, Jiaxing University, Jiaxing, Zhejiang, China; ^6^ School of Medicine, Tongji University, Shanghai, China

**Keywords:** fetal growth restriction (FGR), Olink proteomics platform, proximity extension assay (PEA), biomarkers, targeted proteomics analysis

## Abstract

**Background:**

Effective intrauterine treatments for placental-mediated fetal growth restriction (FGR) remain limited, necessitating reliable protein biomarkers for early diagnosis and management.

**Methods:**

In this study, we analyzed differential protein expression in peripheral blood plasma samples from 44 placental-mediated FGR patients and 44 normal pregnant women using the Olink-Explore-384-Inflammation panel. The analysis identified significant differences in protein expression levels, followed by enrichment analyses to explore the underlying biological mechanisms. Protein-protein interaction (PPI) network analysis and Least Absolute Shrinkage and Selection Operator (LASSO) modeling were used to identify key proteins as potential biomarkers.

**Results:**

We identified 225 proteins with significantly altered expression between FGR patients and normal pregnancies. Proteins such as Placental Growth Factor (PGF) and Hepatocyte Growth Factor (HGF) were previously found to be strongly associated with FGR. In addition, we discovered novel proteins potentially associated with FGR, including ESM1 and TIMP3. Enrichment analyses revealed that several pathways, including placental dysfunction, inflammatory responses, and oxidative stress, may play crucial roles in FGR pathophysiology. PPI network analysis further identified key proteins such as ANGPT2, CD40, and HGF, as potentially linked to FGR. LASSO modeling validated PGF and ESM1 as important biomarkers. Additionally, integrating a multi-protein panel with blood flow disruption analysis significantly improved diagnostic accuracy.

**Conclusion:**

Our findings provide valuable insights into the molecular mechanisms of FGR, identifying key proteins as potential biomarkers. The multi-protein panel model offers a promising tool for early screening and diagnosis of FGR.

## 1 Introduction

Fetal Growth Restriction (FGR) occurs when a fetus fails to achieve its genetically determined growth potential within the uterus, manifesting as a fetal weight below the 10th percentile for gestational age (1). The etiology of FGR is complex, placental-mediated FGR is the most prevalent subtype. It holds the greatest promise for improving adverse outcomes through clinical prevention and management strategies (2). As the mechanism of placental-mediated FGR is unknown and there is a lack of effective clinical screening, prevention, diagnosis and intervention, related research has become a hotspot of concern at home and abroad (3). Placental-mediated FGR is associated with several pathological states of pregnancy, with the mother leading to the development of preeclampsia and the fetus showing growth restriction, poses substantial risks to both maternal and fetal health (4). While the global incidence of FGR varies is approximately 5%-10%, it can exceed 30% among pregnant women with preeclampsia (5). The clinical manifestations of FGR are often subtle and typically diagnosed through routine prenatal check-ups and ultrasound examinations (6). Pregnant women may exhibit symptoms of preeclampsia, such as gestational hypertension and proteinuria (7). During ultrasound examinations, fetal growth indicators such as biparietal diameter, abdominal circumference, and femur length are significantly smaller for the corresponding gestational age. Additionally, decreased fetal biophysical scores and reduced amniotic fluid also signal FGR (8). FGR not only affects fetal growth and development but may also lead to a series of adverse pregnancy outcomes. Infants born with FGR may experience low birth weight, neonatal asphyxia, neonatal death, and in the long term, may face intellectual developmental delays, growth retardation, and an increased risk of metabolic diseases in adulthood (9). Therefore, early diagnosis and intervention for FGR are crucial for improving maternal and fetal outcomes.

Current intervention strategies based on abnormal placental-mediated FGR phenotypes are extremely limited, with the underlying reason being a lack of understanding of placental-mediated FGR etiological mechanisms. It is currently believed that the placenta is the organ of origin for placental-mediated FGR pathogenesis, with placental development, cellular communication at the maternal-fetal interface, and the placenta-fetal gut axis being the key events surrounding placental-mediated FGR etiological mechanisms (10). Extensive studies have explored potential biomarkers for FGR, including placental hormones, inflammatory cytokines, and metabolites (11). However, individual biomarkers often lack sufficient sensitivity and specificity (12). Noteworthy biomarkers include human placental lactogen (hPL) (13) and pregnancy-associated plasma protein A (PAPP-A) (14), whose decreased levels correlate with FGR. Elevated Inflammatory cytokines like interleukin-6 (IL-6) (15) and tumor necrosis factor-α (TNF-α) (16) reflect placental inflammation and oxidative stress. Additionally, alterations in metabolites such as placental growth factor (PlGF) (17) and soluble fms-like tyrosine kinase-1 (sFlt-1) (18) have been studied to assess placental function and predict FGR risk.

However, current research still faces several unresolved problems. Firstly, existing biomarkers often lack adequate sensitivity and specificity for broad clinical use (19). Secondly, FGR is multifactorial, including maternal, fetal, and placental factors, complicating comprehensive assessment of FGR through single biomarkers (20). Lastly, early diagnosis of FGR remains challenging due to its nonspecific nature of early symptoms and the limitations of ultrasound examinations (21).

Until a validated single screening indicator or model is established, both domestic and international guidelines do not recommend clinical screening for placental-mediated FGR in isolation (22). Currently, the majority of clinical studies rely on early-pregnancy preeclampsia screening models or first- and second-trimester Down syndrome serum screening models to predict FGR. Except for placental growth factor (PlGF), which has a sensitivity of 27%, most single biomarker screenings exhibit low sensitivity and limited value (14). With the widespread adoption of noninvasive prenatal testing, the establishment of predictive models for early-onset severe placental-mediated FGR during the first trimester, based on fetal free DNA and RNA derived from maternal plasma, combined with novel biomarkers such as SPINT1 and Ang2, represents a focal area of future clinical research (23, 24).

To address these challenges, the objective of our research is to identify differentially expressed proteins as potential biomarkers for placental-mediated FGR through the application of advanced targeted proteomics technology, specifically the OLINK technology. In recent years, this technology has gradually been applied in research, offering the potential to discover biomarkers through large-scale screening of protein changes in maternal plasma (25). However, there are currently limited examples of OLINK technology’s application in FGR research, which represents an innovative aspect of our study. The FGR cases included in this project are all attributed to placental perfusion insufficiency, and we have initially established a precise diagnostic process for placental-mediated FGR: this involves screening for fetal factors (genetic, structural, infectious, etc.) and assessing ultrasound and maternal blood biomarkers related to placental function (26). Our inclusion criteria are stringent compared to previous basic and clinical studies. Ultimately, we aim to identify novel FGR biomarkers with higher sensitivity and specificity. This will facilitate early diagnosis, risk assessment, and individualized treatment of FGR, ultimately improving maternal and fetal outcomes.

## 2 Methods

### 2.1 Patient sample collection

**Figure 1:**
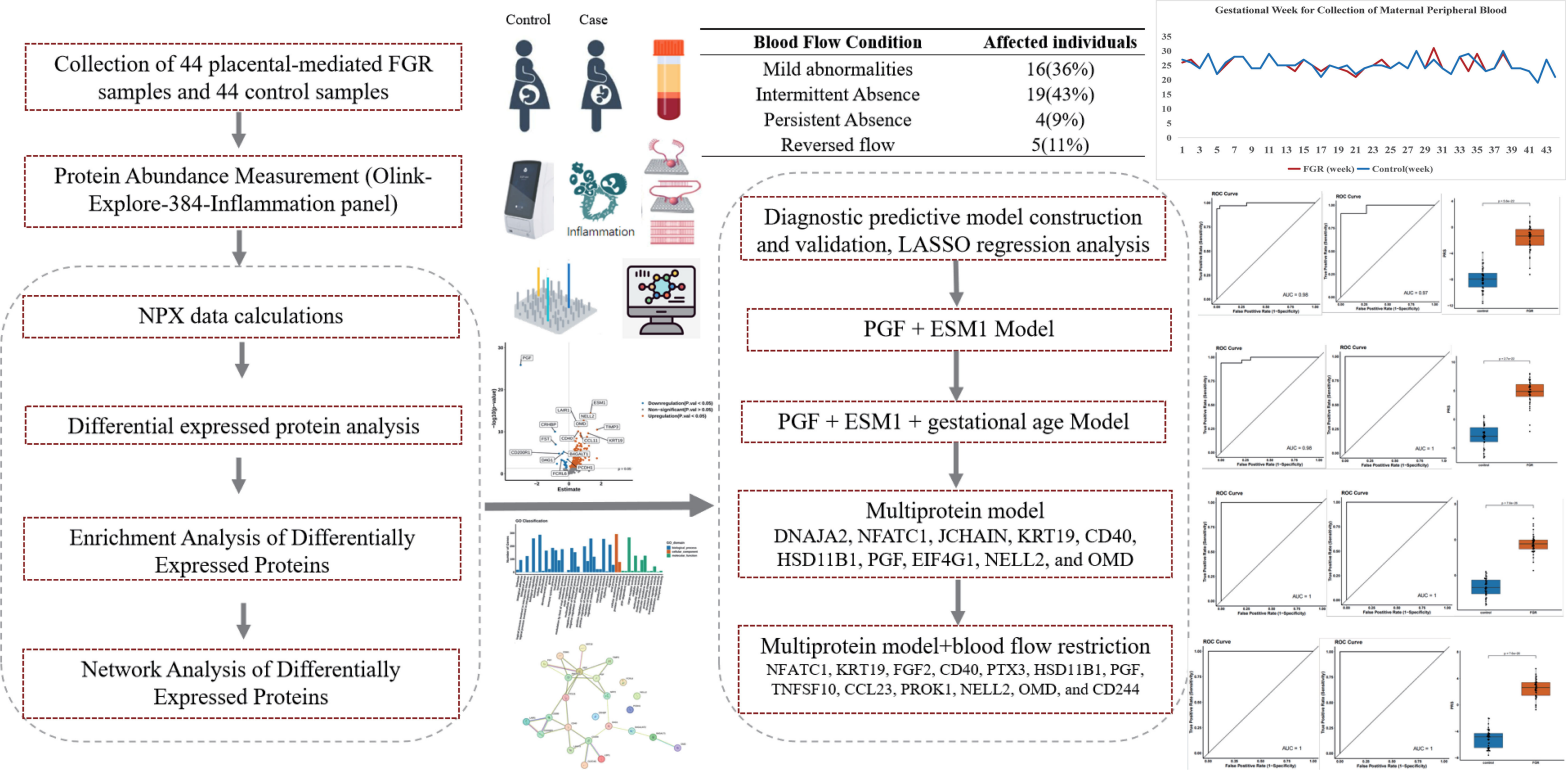
Workflow of the study. The analytical workflow and key findings are presented in this figure. We analyzed differential protein expression in peripheral blood plasma samples from 44 placental-mediated FGR patients and 44 normal pregnant women using the Olink-Explore-384-Inflammation panel. The analysis identified significant differences in protein expression levels, followed by enrichment analyses to explore the underlying biological mechanisms. PPI network analysis and LASSO modeling were used to identify key proteins as potential biomarkers.

From 2019 to 2024, we conducted a retrospective and prospective study at Shanghai First Maternity and Infant Hospital, enrolling 44 patients with placental-mediated FGR and 44 normal controls. Inclusion criteria for placental-mediated FGR patients included an Abdominal Circumference (AC)/Estimated Fetal Weight (EFW) ratio below the 3rd percentile for gestational age, abnormal blood flow, negative genetic testing, and a gestational age ranging from 19 to 31 weeks (with a mean of 25 weeks). Exclusion criteria included multiple gestations and fetal structural abnormalities. Control participants were matched for gestational age with the FGR group and excluded for any fetal/placental abnormalities and abnormal noninvasive prenatal test results. All participants provided written informed consent, and the study was approved by the Ethics Committee of Shanghai First Maternity and Infant Hospital (Approval Number: KS20262). The workflow of this study is illustrated in **Figure 1**.

Peripheral blood samples (2 mL) were collected from each participant. Plasma samples were obtained by centrifugation at 1200g for 15 minutes, processed to remove cellular debris, fats, and other impurities, and then stored at -80°C for subsequent analysis.

### 2.2 Protein abundance measurement

Plasma samples were analyzed for 368 inflammation-related proteins using the Olink-Explore-384-Inflammation panel (Olink™ Proteomics, Uppsala, Sweden) with Proximity Extension Assay (PEA) technology on the Illumina NGS sequencing platform. The procedure involved three main steps: incubation (where plasma samples interacted with 368 antibody pairs tagged with unique DNA oligonucleotides), extension (amplification and enrichment of DNA fragments), and detection (sequencing of enriched DNA fragments for data collection).

Each detection panel included two quality control (QC) systems. The internal QC system monitored amplification using an Immuno control, an Extension control, and a Detection control, while external QC system consisted of eight references (two sample controls, three negative controls, and three inter-plate controls) for data normalization. The QC criteria required that the median value of the negative controls remain within five standard deviations of predefined value set for each experiment and each sample’s average count exceeded 500.

### 2.3 NPX data calculations

Sequencing data were converted into counts files using Olink’s bcl2count software, and processed through the NPX Explore Software to obtain the Normalized Protein Expression (NPX) values on a log2 scale. We used Z-score normalization, calculated as: NPXnorm=NPXi−mean(NPX)/SD(NPX). Tools like ComBat from the ‘sva’ R package was utilized to correct them. The NPX calculation formula, ExtNPX_i,j_ = log2 (counts (sample_j_Assay_i_)/counts (ExtCtrl_j_)) (where i represents a specific assayed protein and j represents a sample), standardizes data using plate controls and applies transformations for normalization across all assays and samples.

Batch effect correction was performed using ComBat to eliminate systematic biases between different experimental batches. The data were analyzed using R software. We utilized data below the Limit of Detection (LOD) as actual usage data, as it could potentially enhance statistical power and reduce false positives.

### 2.4 Differential protein analysis

Differentially Expressed Proteins (DEPs) between different groups were identified using the R-package OlinkAnalyze (version 3.3.1). Based on variance homogeneity and normality testing, we employed either a t-test or Wilcoxon rank-sum test to compare protein levels between FGR patients and controls. Plasma proteins with FDR-adjusted *p*-values less than 0.05 were considered as differentially expressed Principal Component Analysis (PCA) was conducted to assess the degree of separation between different groups. Visualizations (boxplots, volcano plots, heatmaps) were created using the R package ‘ggplot2’.

### 2.5 Enrichment analysis and network analysis

All genes with p-values less than 0.05 were utilized for enrichment analyses for DEPs included GO, KEGG pathway, and COG enrichment analyses using R packages. PPI analysis was conducted focused on the top 10 upregulated and top 10 downregulated proteins using the STRING database (STRING: functional protein association networks (string-db.org)) to investigate the biological processes underlying protein interactions.

### 2.6 Diagnostic predictive model construction and validation

To establish a predictive model distinguishing FGR patients, samples were split into training and test sets (3:1 ratio) using the caret R package (27). LASSO regression analysis was conducted using the glmnet R package (28) (29). The training set samples were trained using a 10-fold cross-validation, and the model hyperparameter lambda with the minimum mean square error (MSE) and corresponding feature set (corresponding modelled protein sets) were selected. The model’s performance was evaluated by Receiver Operating Characteristic (ROC) curves, sensitivity, specificity, and AUC values in the test set using the pROC R package (30).

A comparison was made between a model incorporating clinical information (gestational age and blood flow restriction phenotype) and a model solely based on protein expression levels to analyze the impact of clinical information on model performance. Another LASSO regression model was established by combining protein features with gestational age. Additionally, a LASSO regression model was constructed by integrating protein features with the phenotype of blood flow disruption.

## 3 Results

### 3.1 Participants clinical information characteristics

**Figure 2:**
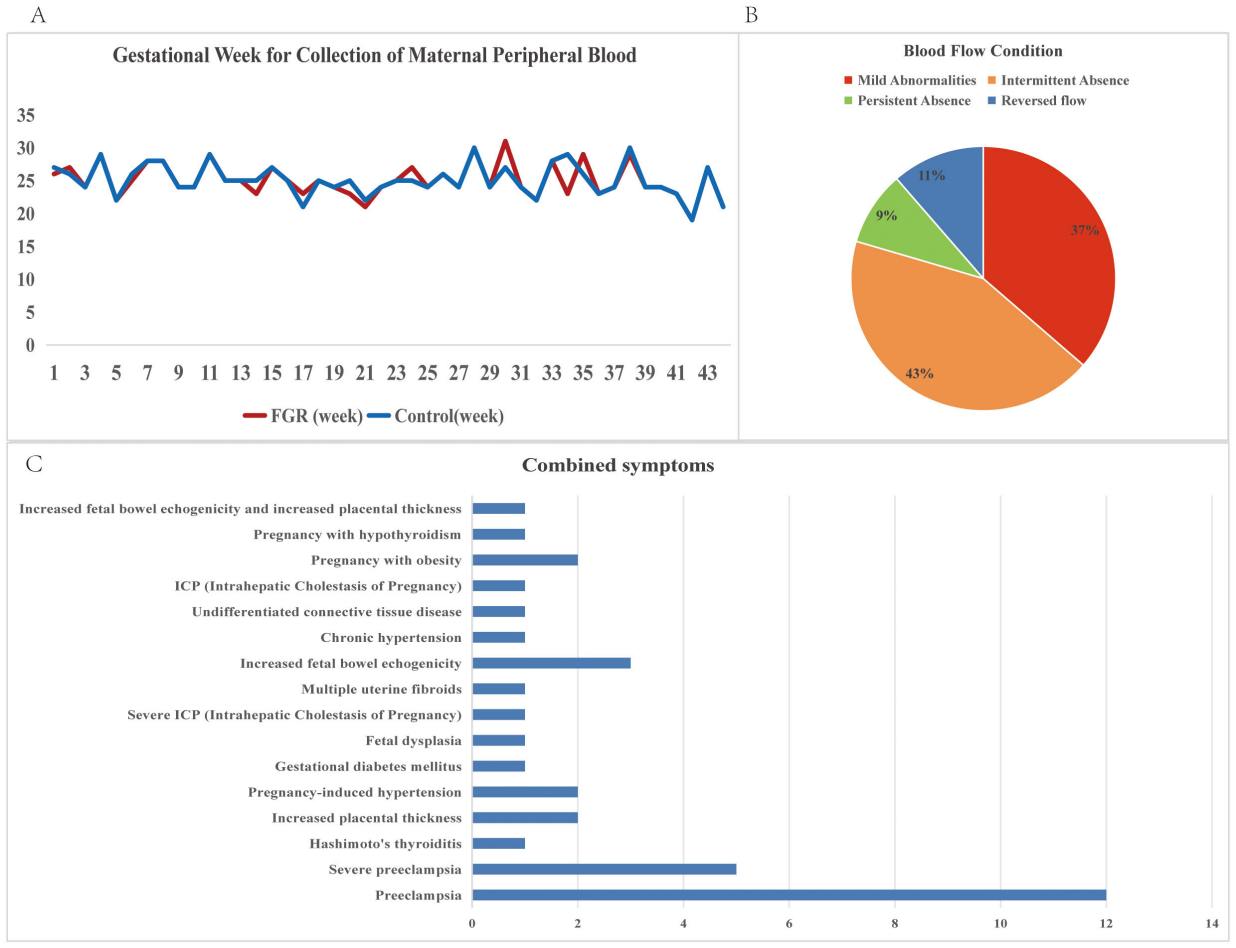
Descriptive statistics of clinical information of FGR patients. **(A)** Gestational Week for Collection of Maternal Peripheral Blood for FGR patients and control samples; **(B)** Blood flow conditions for FGR patients; **(C)** Combined symptoms for FGR patients.

This study enrolled a total of 44 patients with placental-mediated FGR and 44 matched controls. **Table 1** presents their clinical details. The mean gestational age at blood collection was 25 weeks, with the control group matched accordingly, as shown in **Figure 2A**. Blood flow abnormalities were common among FGR patients, with 36% showing mild abnormalities (Doppler changes (UA PI >95th percentile, UtA PI >95th percentile, absent or reversed end-diastolic velocity) 100%), with 52% showing end-diastolic umbilical artery blood flow issues (43% with intermittent ischemia and 9% with persistent ischemia), while 11% exhibited reversed end-diastolic flow (**Figure 2B**).

**Table 1:** Clinical characteristics of placental-mediated FGR patients and the healthy controls.

Clinical information	Patients	Controls
Cohort size, n	44	44
Amniocentesis Gestational Week (Blood Collection Gestational Week)	25 + 2	25 + 2
Blood Flow Condition	Mild abnormalities (Doppler changes(UA PI >95th percentile, UtA PI >95th percentile, absent or reversed end-diastolic velocity)100%) (36%); Intermittent Absence (43%); Persistent Absence (9%); Reversed flow (11%).	Normal (100%)
Medical History Remarks	Preeclampsia (27%); Severe preeclampsia (11%); Hashimoto’s thyroiditis (2%); Increased placental thickness (5%); Pregnancy-induced hypertension (5%); Gestational diabetes mellitus (2%); Fetal dysplasia (2%); Severe ICP (Intrahepatic Cholestasis of Pregnancy) (2%); Multiple uterine fibroids (2%); Increased fetal bowel echogenicity (7%); Chronic hypertension (2%); Undifferentiated connective tissue disease (2%); ICP (Intrahepatic Cholestasis of Pregnancy) (2%); Pregnancy with obesity (5%); Pregnancy with hypothyroidism (2%); Increased fetal bowel echogenicity and increased placental thickness (2%)	Normal (100%)

Complications and disease states were noted, with preeclampsia (38%, including 11% severe cases) being the most prevalent, followed by placental thickening, gestational hypertension, and enhanced fetal intestinal echogenicity (all at 7%) (**Figure 2C**).

### 3.2 Protein detection and differential expression analysis

**Figure 3:**
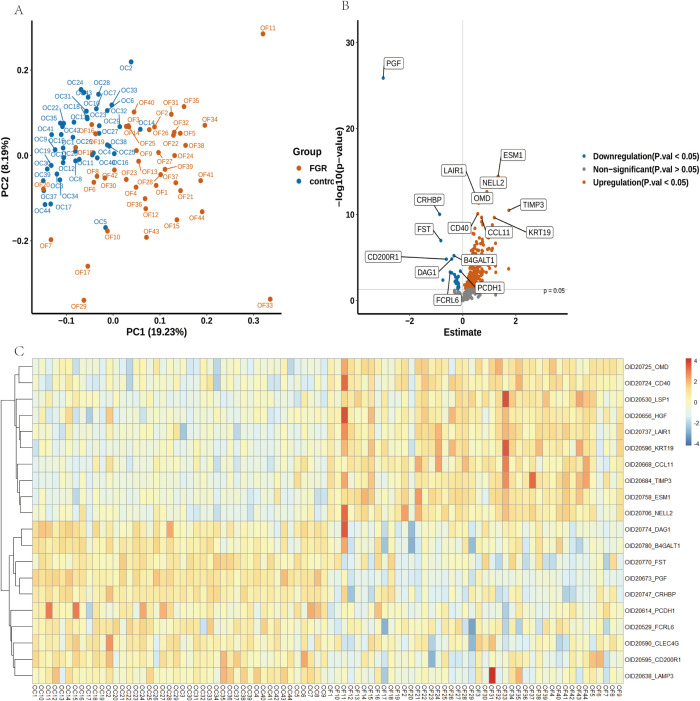
Alterations in Plasma Proteins in FGR Patients Compared to Controls. **(A)** A Principal Component Analysis (PCA) plot illustrates the distribution of samples from 44 FGR patients (depicted in yellow) and 44 control donors (depicted in blue). **(B)** A volcano plot visualizes the log2 fold changes and -log10 (FDR-adjusted p-values) of 368 proteins in FGR samples compared to controls. The size of the points is proportional to the log10 (FDR-adjusted p-value), with the top 8 downregulated (blue) and upregulated (red) proteins highlighted. The FDR-adjusted p-value threshold of 0.05 is indicated by a dashed red line. **(C)** A heatmap depicting protein level changes between FGR and control groups; only the top 10 upregulated and downregulated proteins are displayed.

We utilized the Olink-Explore-384-Inflammation panel to analyze 368 proteins in plasma samples from FGR patients and controls. PCA plot illustrates distinct distribution of plasma samples (**Figure 3A**). Differential analysis identified 225 proteins with significant differences (P ≤ 0.05), of which 200 were upregulated and 25 were downregulated (FDR-corrected P ≤ 0.05) (**Figure 3B**). PGF (P-value: 1.33E-26) exhibiting the most significant down regulation, ESM1 (P-value: 3.71E-15) was significantly up regulated. Heat map of the top 10 upregulated and downregulated proteins both groups are shown in **Figure 3C**. **Table 2** provides corresponding NPX data and p-values, showing significant proteomic differences in FGR patients compared to the control group.

**Table 2:** Top 10 up regulated and down regulated DEPs of placental-mediated FGR patients and the healthy controls (P<0.05).

Protein	NPX in FGR	NPX in control	P.value	Up/Down
OID20530_LSP1	0.139172727	-0.614604545	1.54e-09	up
OID20725_OMD	1.939686364	1.173961364	1.34e-12	up
OID20724_CD40	0.440115909	-0.127293182	8.02e-11	up
OID20668_CCL11	-0.294215909	-1.004059091	2.14e-10	up
OID20737_LAIR1	-0.689518182	-1.290847727	4.55e-12	up
OID20596_KRT19	1.456854545	0.268697727	2.27e-10	up
OID20656_HGF	1.074422727	0.365086364	6.91e-10	up
OID20684_TIMP3	2.984511364	1.245163636	3.24e-11	up
OID20758_ESM1	1.773231818	0.438634091	3.71e-15	up
OID20706_NELL2	1.028463636	0.119579545	2.22e-13	up
OID20770_FST	1.872045455	2.697547727	1.08e-07	down
OID20673_PGF	1.829329545	4.828772727	1.33e-26	down
OID20747_CRHBP	-0.315909091	0.555163636	9.54e-11	down
OID20774_DAG1	1.230606818	1.642861364	1.47e-05	down
OID20780_B4GALT1	1.401286364	1.733475	6.08e-06	down
OID20590_CLEC4G	0.07615	0.384172727	9.76e-04	down
OID20595_CD200R1	-0.096979545	0.519045455	1.55e-05	down
OID20638_LAMP3	-1.214970455	-0.804790909	6.16e-04	down
OID20614_PCDH1	0.053875	0.143943182	3.79e-04	down
OID20529_FCRL6	1.006875	1.481004545	4.96e-04	down

NPX, Normalized protein expression data, which were relative and log2 transformed.

### 3.3 Enrichment analysis of differentially expressed proteins

**Figure 4:**
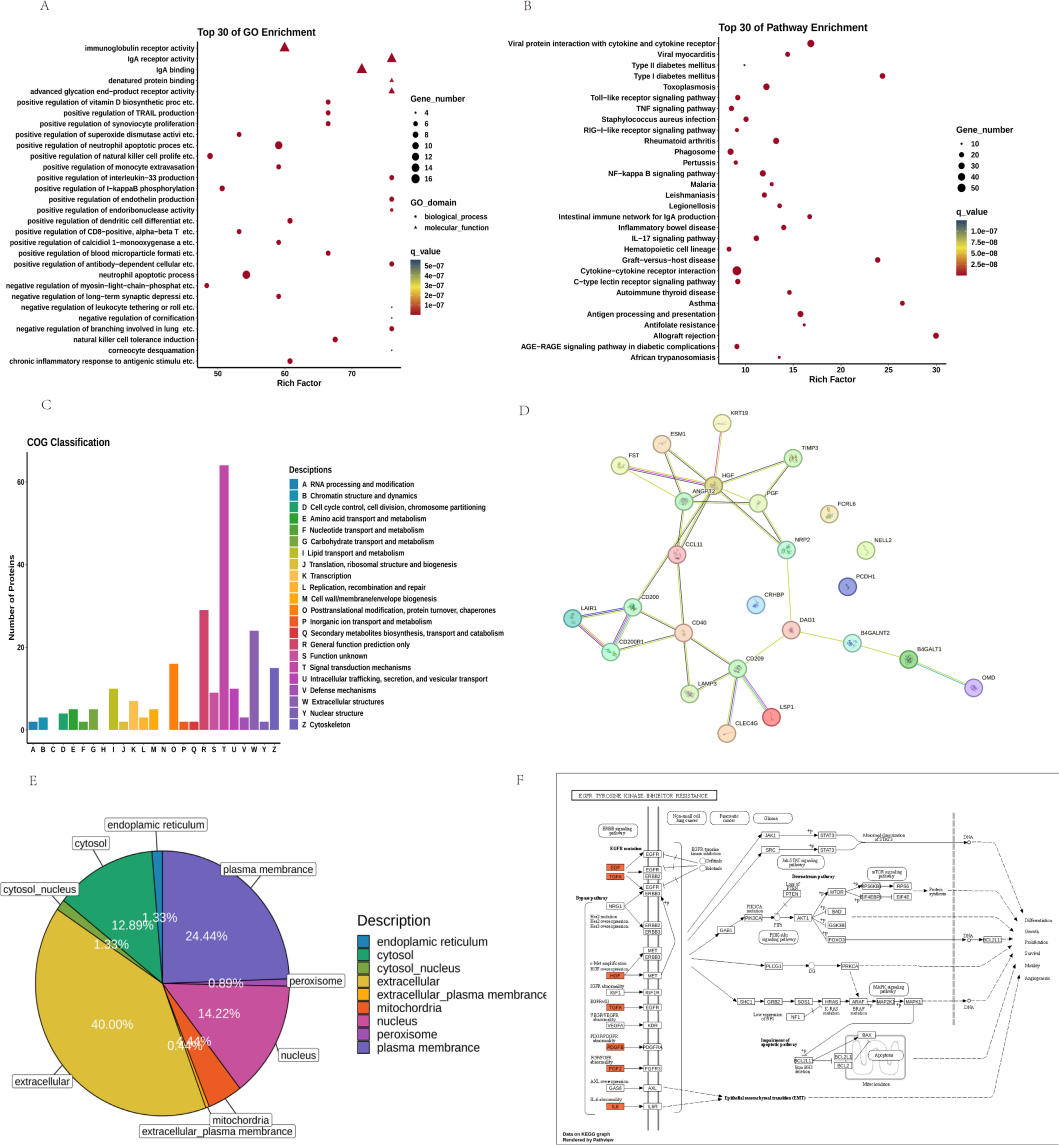
Analysis of the Top 30 Differential Proteins between FGR and Control Groups: **(A)** GO Pathways and **(B)** KEGG Pathways; **(C)** COG Classification; **(D)** PPI Interaction Network Plot for the Top 10 Upregulated and Downregulated Proteins; **(E)** Subcellular Localization; **(F)** Path View of EGFR Tyrosine Kinase Inhibitor Resistance.

GO and KEGG enrichment analyses of the 225 differentially expressed proteins revealed key pathways. The top 30 GO terms (**Figure 4A**) included Positive Regulation of Endothelin Production (P-Value: 9.66E-12), Positive Regulation of Monocyte Extravasation (P-Value: 3.56E-11), Positive Regulation of Superoxide Dismutase Activity (P-Value: 6.34E-10).

KEGG pathway analysis (**Figure 4B**) identified several signaling pathways potentially relevant to FGR, including AGE-RAGE Signaling Pathway in Diabetic Complications (P-value: 1.74E-13), NF-κB (P-value: 3.98E-20), IL-17 (P-value: 5.21E-17), and TNF Signaling Pathway (P-value: 1.74E-13). These pathways underscore mechanisms like inflammation, immune regulation, and oxidative stress as central to FGR pathology.

COG analysis (**Figure 4C**) categorized DEPs based on cellular metabolism, signal transduction, cell cycle control, and immune responses.

### 3.4 Network analysis of differentially expressed proteins

PPI analysis (**Figure 4D**) identified hub proteins, including ANGPT2, CD40, NRP2 and HGF.

Subcellular Location analysis (**Figure 4E**) revealed mitochondrial-associated proteins (14.22%) and extracellular proteins (40.00%) as significant increase, suggesting altered energy metabolism and extracellular signaling in FGR.

### 3.5 FGR prediction model and calculation of disease prediction rates

**Figure 5:**
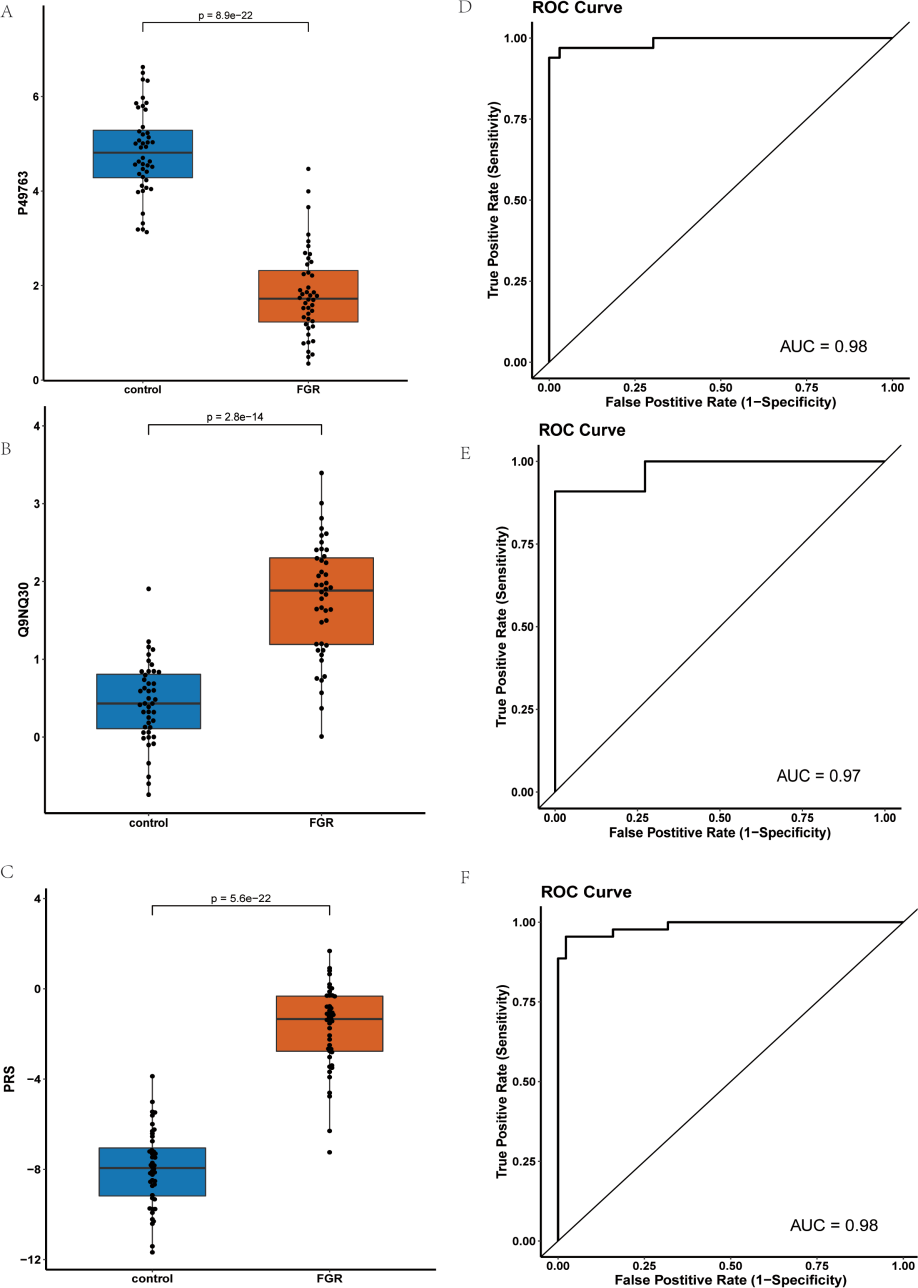
Comparison and Analysis of PGF and ESM1 Proteins in Control and FGR Groups: **(A)** Expression Levels of PGF Protein in Control and FGR Groups; **(B)** Expression Levels of ESM1 Protein in Control and FGR Groups; **(C)** Comparison of PRS Risk Scores between Control and FGR Groups after Modeling with Combined PGF and ESM1; **(D)** ROC Curve of the PGF and ESM1 Model in the Training Set; **(E)** ROC Curve of the PGF and ESM1 Model in the Test Set; **(F)** ROC Curve of the PGF and ESM1 Model in the Inflammatory Panel Protein Set.

Using LASSO, we constructed a prediction model consisting of PGF and ESM1 (**Figure 5**). PGF (P-value: 1.33E-26) had the smallest p-value among downregulated proteins) while ESM1(P-value: 3.71E-15) was the most significant upregulated protein.

**Figure 5A** presents the NPX values of PGF in FGR patients compared to controls, demonstrating significant down regulation of PGF in FGR patients. Similarly, **Figure 5B** shows the NPX values of ESM1, revealing a significant upregulation of ESM1 in FGR patients. **Figure 5C** compares the Protein Risk Score (PRS) differences between FGR and control groups modeled using the combination of PGF and ESM1, indicating significantly higher PRS for the FGR group.

**Figures 5D** and **4E** demonstrate that the PGF and ESM1 model effectively distinguished between FGR patients and normal controls in both the discovery and validation sets, achieving Area Under the Curve (AUC) values of 0.98 and 0.97, respectively. **Figure 5F** displays the Receiver Operating Characteristic (ROC) curve of the combined PGF and ESM1 model within an inflammatory protein panel, yielding an AUC value of 0.98.

To elucidate the impact of gestational age at sampling on diagnostic performance, we incorporated gestational age into a new LASSO-based model. This model exhibited an AUC value of 0.98 in the discovery cohort and 1.0 in the validation set, suggesting its effectiveness as a diagnostic tool for FGR.

We then constructed a diagnostic model using the 225 differentially expressed proteins, selecting a model with the minimum number of proteins (10) after 100 rounds of cross-validation. The final set included DNAJA2, NFATC1, JCHAIN, KRT19, CD40, HSD11B1, PGF, EIF4G1, NELL2, and OMD. This model achieved AUC values of 1 in both the training and validation sets, with significant PRS differences and significantly higher scores for FGR patients compared to controls.

Given the importance of the blood flow restriction as a key phenotype in FGR, we developed another model incorporating blood flow restriction alongside the 225 proteins. After 100 rounds of cross-validation, we selected a model with 13 proteins plus one blood flow restriction indicator (NFATC1, KRT19, FGF2, CD40, PTX3, HSD11B1, PGF, TNFSF10, CCL23, PROK1, NELL2, OMD, and CD244). This model also achieved perfect AUC values in both the training and validation sets, demonstrating significant PRS differences and significantly higher scores for FGR patients.

## 4 Discussion

FGR is a major cause of intrauterine fetal demise in late pregnancy and is associated with profound long-term health risks, including increased susceptibility to metabolic disorders such as metabolic syndrome, diabetes, and hypertension in adulthood (1). Due to the limited intrauterine treatments options currently available, identifying reliable protein biomarkers for early FGR diagnosis and management is critical (5). In this study, we utilized the Olink-Explore-384-Inflammation panel to analyze peripheral blood plasma from 44 placental-mediated FGR patients and 44 matched controls, revealing 225 differentially expressed proteins.

Key findings include PGF and HGF, which are well-documented in their associations with FGR. PGF, critical for placental angiogenesis, was notably downregulated, consistent with previous studies linking its reduced levels to poor uterine spiral artery remodeling and placental hypoperfusion in FGR (31, 32). Brouillet et al. (2012) demonstrated that impaired PGF signaling leads to placental developmental abnormalities and fetal hypoperfusion, making it a strong predictor of FGR (33) (34). HGF is another factor closely related to cell growth, motility, and angiogenesis (35). Somerset et al. (1997) found that abnormal HGF signaling results in placental hypoplasia, which is a critical factor in FGR (36). Research reports have indicated that lower HGF levels are associated with impaired trophoblast invasion and reduced placental vascularization, leading to fetal growth restriction (37).

In addition to these established markers, we identified two novel potential protein biomarkers: ESM1 and TIMP3. ESM1(Endothelial Cell-Specific Molecule-1), is a dermatan sulfate proteoglycan. ESM1 plays a pivotal role in the pathogenesis of several cancers, with its overexpression closely associated with tumor growth, progression, and angiogenesis (38). In our research, ESM1 was significantly upregulated in MVM-FGR samples. Considering ESM1’s functions in cell proliferation, migration, and angiogenesis, there may be a correlation between ESM1 and the occurrence and development of FGR, though this requires further validation. TIMP3(Tissue Inhibitor of Metalloproteinase 3), functions as an inhibitor of inflammatory cytokines. It negatively regulates TACE (TNF-α Converting Enzyme), thereby influencing the release of TNF-α (Tumor Necrosis Factor-α). This regulatory effect, in turn, can impact the body’s inflammatory response and vascular remodeling processes (39). In our study, TIMP3 was upregulated in patients, suggesting that abnormal expression of TIMP3 may affect blood flow in the placenta and umbilical cord, hindering the fetus’s ability to obtain adequate nutrition from the mother, potentially leading to FGR. However, this also necessitates further verification. Collectively, these proteins hold promise as potential biomarkers for placental-mediated FGR screening.

Functional enrichment analyses (GO, KEGG, and COG) provided insights into the pathways associated with FGR. The GO analysis highlighted pathways such as the positive regulation of endothelin production pathway involves endothelin, a potent vasoconstrictor, which plays a crucial role in regulating placental blood flow (40), affecting the nutrient and oxygen supply to the fetus, and consequently resulting in FGR (41). Similarly, the positive regulation of monocyte extravasation is associated with chronic placental inflammation, where monocytes may play a significant role in the immune regulation of the placenta, impacting fetal growth (42). The positive regulation of CD8+ T-cell proliferation pathway is related to immune responses, and abnormal placental immune function is a common feature of FGR (43); the proliferation and regulation of T-cells may affect placental health and fetal growth (44). KEGG pathway analysis identified pathways like AGE-RAGE signaling pathway, implicated in oxidative stress and inflammation, and NF-κB and IL-17 signaling pathways (45). The NF-κB signaling pathway, an important inflammatory and immune regulatory pathway, may affect fetal growth through the induction of placental inflammation via its abnormal activation (46). The IL-17 signaling pathway is associated with proinflammatory responses and may exacerbate placental inflammation, influencing the pathogenesis of FGR (47).

A logistic regression model using LASSO regression (48) was employed to identify key protein biomarkers for differentiating placental-mediated FGR patients from controls. The model, which incorporated PGF and ESM1, achieved AUC values of 0.98 and 0.97 in discovery and validation sets, respectively, demonstrating high diagnostic accuracy. Incorporating gestational age further enhanced model performance, achieving an AUC of 0.98 in the discovery set and 1.0 in the validation set. Our research not only validated the importance of PGF and ESM1 as potential biomarkers for placental-mediated FGR but also significantly improved the diagnostic accuracy of the model through multi-protein combination models and the inclusion of blood flow disruption analysis.

This research project has made meaningful progress in exploring protein biomarkers for placental-mediated FGR, with notable advantages: All cases of placental-mediated FGR included were specifically caused by placental perfusion insufficiency, confirmed through prenatal ultrasound blood flow assessments and postpartum pathological examinations, while excluding genetic and infectious causes. Compared to previous basic and clinical studies, the inclusion criteria were rigorously defined. This is the first time that the Olink technology has been employed to analyze human samples of placental-mediated FGR.

While our study provides novel insights into FGR-associated biomarkers, several limitations must be acknowledged. First, although this study included 44 patients with placental-mediated FGR and 44 normal pregnant women, the sample size remains relatively small. This limitation may hamper statistical power, increase the risk of false positives or false negatives, and may not fully reflect the heterogeneity in the placental-mediated FGR patient population. Future studies with larger cohorts are essential to validate our findings and improve the generalizability of these biomarkers. If increasing sample size is not feasible, meta-analyses integrating multiple datasets could enhance robustness. Secondly, our study samples are based on the Chinese population, which may introduce population-specific biases. Regional, ethnic, and socioeconomic factors could influence plasma protein expression, and expanding research to multi-ethnic cohorts is necessary to assess cross-population validity. Currently, there are no international studies identifying ESM1 and TIMP3 as biomarkers for placental-mediated FGR, highlighting the innovative nature of our research. We are going to lead a global multicenter study, we have sufficient resources to conduct this research. The plan is to initially conduct a small-scale clinical cohort study domestically; if the results are promising, we will proceed with an international multicenter study. Thirdly, our findings are primarily based on statistical correlations rather than direct causal relationships. Functional validation of PGF, HGF, ESM1, and TIMP3 through *in vitro* trophoblast models and *in vivo* animal studies is crucial for confirming their mechanistic roles in FGR pathogenesis. If experimental validation is not feasible, literature-based discussion on their biological functions should be expanded to provide further mechanistic insights. Additionally, longitudinal studies tracking biomarker expression at different gestational stages would help assess their predictive value. Independent replication in different clinical settings will also confirm the robustness of these biomarkers. If longitudinal studies are currently unfeasible, discussing the importance of time-series analyses in biomarker evaluation can strengthen future research directions. Comparative studies including patients with other placental disorders, such as preeclampsia, could help distinguish FGR-specific markers from general placental dysfunction markers. If such comparisons are not feasible, future studies should aim to delineate disease-specific biomarker signatures through comparative analyses. Lastly, our pathway analysis identified NF-κB and IL-17 signaling pathways as potentially relevant to FGR. A more detailed discussion on how these pathways may serve as therapeutic targets could add clinical value to our findings. Further research should explore pharmacological interventions targeting these pathways to mitigate FGR progression. If experimental validation is not available, reviewing existing therapies targeting these pathways and their potential applications in FGR treatment could be an informative addition.

## 5 Conclusion

In conclusion, this study utilized Olink proteomic analysis to PGF, HGF ESM1 and TIMP3 as key biomarkers for placental-mediated FGR. Biological pathway enrichment analysis underscores the significance of placental vascular function, immune regulation, and oxidative stress in the development of placental-mediated FGR. Our combined PGF and ESM1 model, alongside a multi-protein panel, demonstrated high demonstrated accuracy with strong sensitivity and specificity, highlighting their potential for clinical application in early detection and risk assessment of placental-mediated FGR.

## Data Availability

The datasets presented in this article are not readily available because the raw data contain potentially identifiable patient information and have not yet been anonymized or processed for deposition in a public repository in compliance with institutional and ethical regulations. Requests to access the datasets should be directed to the corresponding author.
