# Correction: Investigation of potential protein biomarkers for the screening of placental-mediated fetal growth restriction disorders using targeted proteomics Olink technology

**DOI:** 10.3389/fimmu.2025.1632381

**Published:** 2025-06-23

**Authors:** Xinyao Zhou, Wuqian Wang, Luan Chen, Yingjun Yang, Xing Wei, Jia Zhou, Kuan Sun, Ping Tang, Xiaofang Sun, Shengying Qin, Luming Sun

**Affiliations:** ^1^ Department of Fetal Medicine & Prenatal Diagnosis Center, Shanghai Key Laboratory of Maternal Fetal Medicine, Shanghai Institute of Maternal-Fetal Medicine and Gynecologic Oncology, Shanghai First Maternity and Infant Hospital, School of Medicine, Tongji University, Shanghai, China; ^2^ Department of Obstetrics and Gynecology, Guangdong Provincial Key Laboratory of Major Obstetric Diseases, Guangdong Provincial Clinical Research Center for Obstetrics and Gynecology, Guangdong-Hong Kong-Macao Greater Bay Area Higher Education Joint Laboratory of Maternal-Fetal Medicine, The Third Affiliated Hospital, Guangzhou Medical University, Guangzhou, China; ^3^ Bio-X Institutes, Key Laboratory for the Genetics of Developmental and Neuropsychiatric Disorders (Ministry of Education), Shanghai Jiao Tong University, Shanghai, China; ^4^ Department of Obstetrics, Center of Fetal Medicine & Intrauterine Pediatrics, Xinhua Hospital, Shanghai Jiao Tong University School of Medicine, Shanghai, China; ^5^ Jiaxing Maternity and Children Health Care Hospital, Affiliated Women and Children Hospital, Jiaxing University, Jiaxing, Zhejiang, China; ^6^ School of Medicine, Tongji University, Shanghai, China

**Keywords:** fetal growth restriction (FGR), Olink proteomics platform, proximity extension assay (PEA), biomarkers, targeted proteomics analysis

In the published article, there was an error in **Figure 2B** as published. Incorrect values were displayed on the pie chart. The corrected **Figure 2** and its caption “Figure 2 Descriptive statistics of clinical information of FGR patients. (A) Gestational Week for Collection of Maternal Peripheral Blood for FGR patients and control samples; (B) Blood flow conditions for FGR patients; (C) Combined symptoms for FGR patients” appear below.

**Figure 2:**
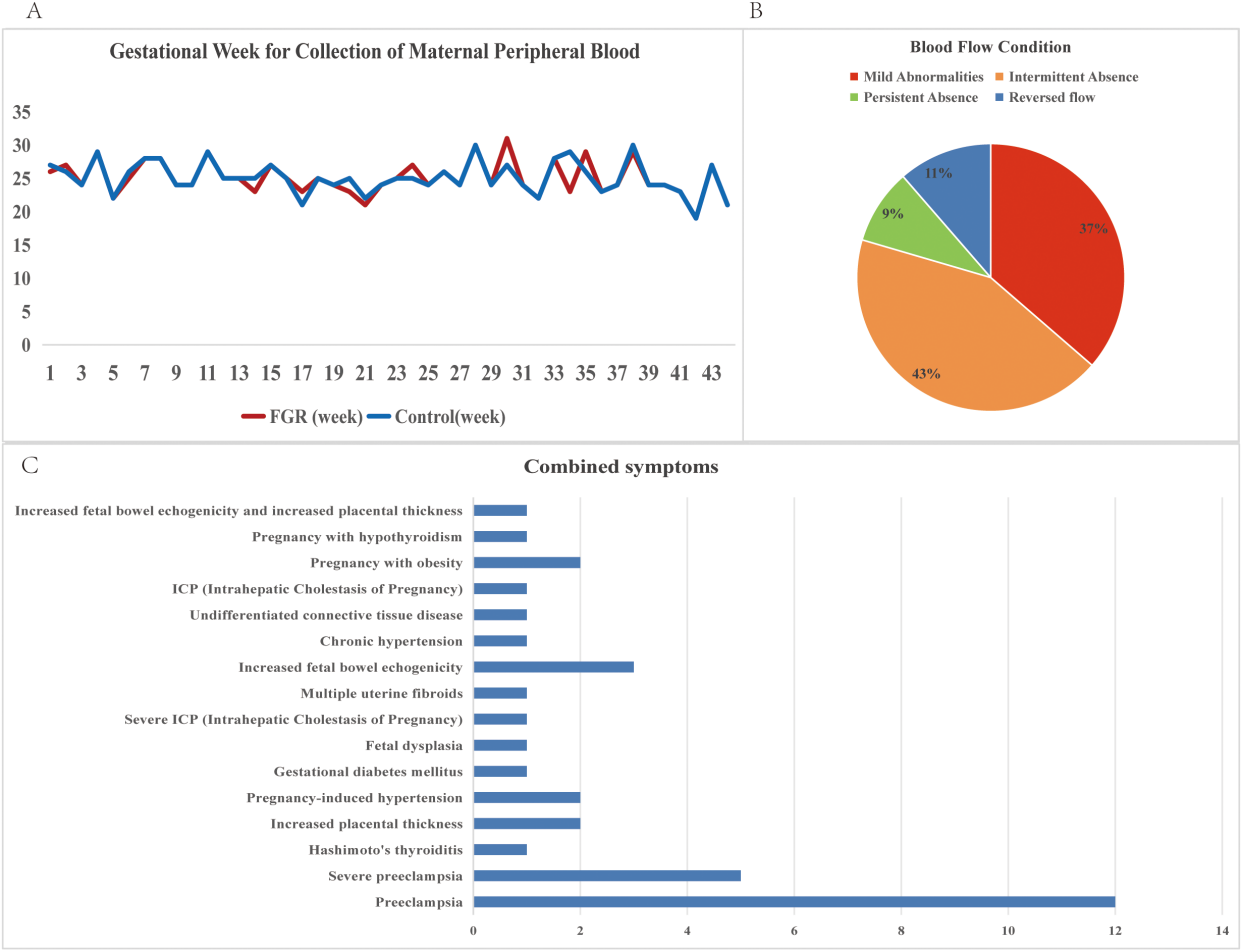
Descriptive statistics of clinical information of FGR patients. **(A)** Gestational Week for Collection of Maternal Peripheral Blood for FGR patients and control samples; **(B)** Blood flow conditions for FGR patients; **(C)** Combined symptoms for FGR patients.

The original article has been updated.

